# ENCOURAGING NEW-BUILD, SMALL, AFFORDABLE, AND ACCESSIBLE HOMES IN AOTEAROA, NEW ZEALAND

**DOI:** 10.1093/geroni/igae098.1328

**Published:** 2024-12-31

**Authors:** Bev James

**Affiliations:** Public Policy & Research Ltd, Wellington, Wellington, New Zealand

## Abstract

The growing need for mainstream housing to be accessible is an international issue, driven by ageing populations and ‘ageing-in-place’ policies. Alongside the impetus to improve housing accessibility, is interest in building smaller dwellings, potentially better suited to an ageing population. Internationally, some jurisdictions mandate accessible standards in new-build housing, while others adopt national voluntary accessibility standards. In Aotearoa New Zealand (NZ) there is no mandatory requirement and few incentives for new-build housing to be accessible. Only an estimated 2% of dwellings are accessible, while one in six people need modifications to live safely in their homes. Our study addresses the dearth of NZ research into achieving accessibility within a small footprint (50m2-75m2). Its focus on new-builds directly addresses a research gap in international literature, which is largely concerned with home modifications rather than new homes built to accessible standards. We report on findings from interviews with housing providers, architects/designers and builders/developers involved in the production of accessible, small homes. Key themes are: accessible design is perceived as good design; awareness, knowledge and understanding of accessible design and its implementation varies; user-centered accessible design is not well understood nor applied; and trade-offs are made between accessibility, affordability and size. Findings contribute to housing policies and practice by identifying levers to improve accessibility in new-build housing. Findings reinforce calls for a multi-pronged approach to achieve housing accessibility, and suggest that “explorative learning” about accessibility is emerging.

